# A Time-Stratified Case-Crossover Study of Ambient Ozone Exposure and Emergency Department Visits for Specific Respiratory Diagnoses in California (2005–2008)

**DOI:** 10.1289/ehp.1409495

**Published:** 2015-12-08

**Authors:** Brian J. Malig, Dharshani L. Pearson, Yun Brenda Chang, Rachel Broadwin, Rupa Basu, Rochelle S. Green, Bart Ostro

**Affiliations:** 1 Air and Climate Epidemiology Section, California Office of Environmental Health Hazard Assessment, Oakland, California, USA; 2 School of Public Health, University of California, Berkeley, Berkeley, California, USA

## Abstract

**Background:**

Studies have explored ozone’s connection to asthma and total respiratory emergency department visits (EDVs) but have neglected other specific respiratory diagnoses despite hypotheses relating ozone to respiratory infections and allergic responses.

**Objective:**

We examined relationships between ozone and EDVs for respiratory visits, including specifically acute respiratory infections (ARI), asthma, pneumonia, chronic obstructive pulmonary disease (COPD), and upper respiratory tract inflammation (URTI).

**Methods:**

We conducted a multi-site time-stratified case-crossover study of ozone exposures for approximately 3.7 million respiratory EDVs from 2005 through 2008 among California residents living within 20 km of an ozone monitor. Conditional logistic regression was used to estimate associations by climate zone. Random effects meta-analysis was then applied to estimate pooled excess risks (ER). Effect modification by season, distance from the monitor and individual demographic characteristics (i.e., age, race/ethnicity, sex, and payment method), and confounding by other gaseous air pollutants were also investigated. Meta-regression was utilized to explore how climate zone–level meteorological, demographic, and regional differences influenced estimates.

**Results:**

We observed ozone-associated increases in all respiratory, asthma, and ARI visits, which were slightly larger in the warm season [asthma ER per 10-ppb increase in mean of same and previous 3 days ozone exposure (lag03) = 2.7%, 95% CI: 1.5, 3.9; ARI ERlag03 = 1.4%, 95% CI: 0.8, 1.9]. EDVs for pneumonia, COPD, and URTI were also significantly associated with ozone exposure over the whole year, but typically more consistently so during the warm season.

**Conclusions:**

Short-term ozone exposures among California residents living near an ozone monitor were positively associated with EDVs for asthma, ARI, pneumonia, COPD, and URTI from 2005 through 2008. Those associations were typically larger and more consistent during the warm season. Our findings suggest that these outcomes should be considered when evaluating the potential health benefits of reducing ozone concentrations.

**Citation:**

Malig BJ, Pearson DL, Chang YB, Broadwin R, Basu R, Green RS, Ostro B. 2016. A time-stratified case-crossover study of ambient ozone exposure and emergency department visits for specific respiratory diagnoses in California (2005–2008). Environ Health Perspect 124:745–753; http://dx.doi.org/10.1289/ehp.1409495

## Introduction

The effects of ozone exposure on respiratory health have long been studied. Both experimental and observational studies have demonstrated ozone’s ability to decrease lung function, incite allergic and inflammatory responses, and promote airway hyperreactivity (Mudway and Kelly 2000; U.S. EPA 2013). These studies implicate oxidative stress pathways as the primary cause, as do studies of other pollutants such as particulates and nitrogen dioxide (NO_2_) (Ayres et al. 2008; Brown 2015; Kelly 2003). Studies have also associated chronic ozone exposures with the onset of asthma and long-term lung function reduction (Fanucchi et al. 2006; Islam et al. 2008). Furthermore, ozone exposure has been linked to increased mortality (Bell et al. 2005), hospitalizations (Ji et al. 2011), primary care physician visits (Yamazaki et al. 2009), and school absences (Gilliland et al. 2001).

Emergency department visits (EDVs) greatly outnumber hospitalizations and can differ noticeably from them by diagnostic composition, demographics, quantity, and temporal characteristics (Winquist et al. 2012). Most ozone-respiratory EDV studies have focused on asthma, yielding a significant relationship when pooled in a review by Ji et al. (2011). However, despite plausible hypotheses connecting ozone with other respiratory outcomes, links to EDVs for non-asthma outcomes have been less evident. Characterizing how ozone exposures relate to these outcomes may help identify important biological mechanisms of airway disease and better delineate the breadth and scale of ozone’s impacts. Furthermore, a number of climate change models predict future increases in ozone related to rising temperatures in a number of regions (Ebi and McGregor 2008). Thus, ozone studies could help illuminate consequences of both near-term and future exposure scenarios, quantify health impacts, and inform important policy decisions.

In this study, we examined ozone exposure and respiratory EDVs in California, a state with both comprehensive records of EDVs and large variations in ozone levels, including areas failing to meet national and state Ambient Air Quality Standards [California Air Resources Board (CARB) 2014]. California has numerous monitors for ozone and other gaseous pollutants, facilitating an examination of confounding by other air pollutants. Additionally, its large, demographically diverse population allows us to explore possible effect modifiers of the relationships we may observe. We utilize these advantages to present an in-depth analysis, examining a population living near ozone monitors to reduce possible exposure misclassification.

## Methods

### Health Outcome Information

The California Office of Statewide Health Planning and Development provided cause-specific emergency visit data for 2005 through 2008 (California Office of Statewide Health Planning and Development 2011). Records were compiled from the Emergency Department (ED) Data and the Patient Discharge Data (PDD), which covered outpatient and inpatient hospital visits, respectively. From PDD records, we included only hospitalizations originating in the ED. Inpatient visits reported date of admission, not ED presentation, so exposures were linked to that date.

We utilized information on each patient’s date of visit, principal diagnosis, residential ZIP code, and subsequent hospitalization (yes/no), as well as information on the patient’s age, race/ethnicity, sex, and expected method of payment. Because data were not linked by person, we were unable to identify multiple visits by the same individual, so each observation was treated as an independent observation. We used the *International Classification of Diseases, 9th Revision* (ICD-9) codes (National Center for Health Statistics, 2011) to identify respiratory visits (ICD-9: 460–519), noting several diagnoses specifically: asthma (ICD-9: 493), pneumonia (ICD-9: 480–486), chronic obstructive pulmonary disease (COPD) (ICD-9: 490–492, 494–496), acute respiratory infections (ARI) (ICD-9: 460–466), and upper respiratory tract inflammation (URTI) (ICD-9: 472–473, 476–477).

The study was approved by the State of California Committee for the Protection of Human Subjects. Informed consent was not required because the data came from anonymous datasets typically used for administrative purposes. Additional safeguards included encryption and electronic and physical barriers to data access.

### Air Pollution and Meteorological Information

Our study used daily 1-hr maximum values for ozone (O_3_), carbon monoxide (CO), nitrogen dioxide (NO_2_), and sulfur dioxide (SO_2_) reported in parts per million by CARB (2011).

**Figure 1:**
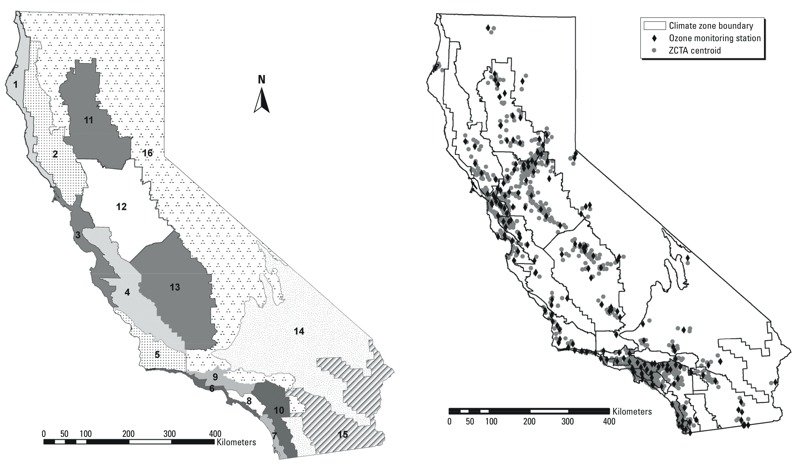
Maps of California CZs and locations of ozone monitors and EDV ZCTA centroids used in this study. CZ boundaries designated by California Energy Commission (2009). Ozone monitor locations provided by CARB (2011).

For greater coverage, we used three meteorology monitoring databases: the California Irrigation Management Information System (California Department of Water Resources 2010), the U.S. Environmental Protection Agency Data Mart (U.S. EPA 2009), and the National Climatic Data Center (2011). From temperature and relative humidity readings, we calculated mean apparent daily temperature in degrees Fahrenheit (°F) (Basu et al. 2008), to account for combined temperature and relative humidity effects. Climate zone (CZ) boundaries were designated by the California Energy Commission to define areas with similar weather, temperature, energy use, and other factors related to climate (Figure 1) (California Energy Commission 2009).

### Study Design

For each EDV, we assigned 1-hr maximum daily air pollution concentrations for O_3_, NO_2_, CO, and SO_2_ from ambient monitors based on ZIP code of residence, requiring that *a*) the air pollution monitor be located within 20 km of the population-weighted centroid of the residential ZIP code, and *b*) the monitor and the ZIP code centroid share the same CZ. The closest monitor was chosen when multiple monitors met such criteria. Temperature monitors were similarly assigned, but with a maximum distance of 10 km. Our aim was to account for California’s diverse climate and geography. Ozone formation is influenced by sunlight and temperature, and locations may not be well represented by nearby monitors if their meteorological conditions differ markedly. Using these restrictions should enhance a monitor’s representativeness of residence-based conditions. Geographical assignment was performed using ArcGIS 9.2 (ESRI 2006) with Hawth’s Tools (Beyer 2008). ZIP code centroids were estimated using U.S. Census 2000 ZCTA (ZIP code tabulation areas) boundaries (Missouri Census Data Center 2006).

We used a time-stratified case-crossover study design (Janes et al. 2005) to compare exposures of individual patients on the day of the EDV to their exposures on up to 4 referent days occurring on the same day of the week during the same month. Because each individual serves as their own control, there is no confounding by factors that do not vary within a month. In addition to classifying exposures for the day of the visit and individual reference days, we also estimated associations with exposures on days before the visit.

### Statistical Analysis

In the first stage of the analysis, we used conditional logistic regression to estimate associations between daily 1-hr maximum ozone (continuous) and each outcome in each of the 16 climate zones, with adjustment for same-day (lag_0_) mean apparent temperature (linear and squared terms), mean apparent temperature for the previous 3 days (lag_13_) (linear and squared terms), and the number of influenza visits during the week by residents of the same county (natural log–transformed after adding 1 to address zero count days). These analyses were performed using the PHREG procedure in SAS 9.3 (SAS Institute Inc. 2000). In the second stage, we estimated an overall effect by combining effect estimates from CZs with a minimum 20 cases in a random effects meta-analysis with the “rmeta” package using R 2.15.3 (Lumley 2012; R Core Team 2008). We report estimated percent excess risks (ER) of EDV per 10-ppb increase in ozone [(odds ratio per 10 ppb – 1) × 100%], and corresponding 95% confidence intervals (CIs). We separately evaluated associations for same-day ozone (lag_0_) and ozone on each of the 3 days prior to the EDV (lag_1_–lag_3_). We also examined the cumulative day associations of ozone for the same- and previous day (lag_01_) and previous 3 days (lag_03_). For each lag, the summation of Akaike’s Information Criteria (AIC) over all the CZs was calculated, with the lowest sum considered best fit for each outcome (Samoli et al. 2008). *I^2^* statistics were also calculated to assess the degree of heterogeneity in each pooled estimate in order to assess the generalizability of our results using the Stata (release 13; StataCorp, College Station, TX) and the “metareg” package (Harbord and Higgins 2008).

Additionally, we conducted analyses specific to warm season (May–October) visits, when ozone levels are generally higher due to increased sunlight catalyzing its formation. We were also interested in possible confounding by other air pollutants (CO, NO_2_, SO_2_), but did not have these values for all of our observations. To investigate this, we first created subsets of data where measurements for ozone and one of these pollutants was available. Then, for the best fitting lag of ozone determined previously, we ran outcome-specific analyses for each co-pollutant subset with ozone alone, and ozone and another pollutant, to allow for comparisons on the exact same population. For co-pollutants, an average of same day and previous day levels (lag_01_) were used. To examine the sensitivity of our COPD definition, we also examined COPD visits specifically for those ≥ 50 years old, and its sensitivity to co-pollutant adjustment as above.

To evaluate potential effect modification, we stratified models by age (< 5, 5–18, 19–64, ≥ 65), race/ethnicity (non-Hispanic Black, non-Hispanic White, Hispanic, non-Hispanic Asian), sex (male, female), and distance from monitor (< 10 km/≥ 10 km). We also stratified by expected primary payment method [private insurance or self-pay/aid (MediCal, self-pay, county, and other indigent programs)] in analyses limited to those < 65 years old to exclude older Medicare recipients. These subsets of our data were analyzed separately through conditional logistic regression and random effects meta-analysis using the same covariates as in the non-stratified models. We then evaluated statistical differences between these stratum-specific pooled estimates by dividing the difference in betas by the pooled standard error to calculate a *z*-score. Significant differences (*p* < 0.10) were identified, using a main comparison group when more than two categories existed. Again, a minimum of 20 cases per CZ after stratification was required to be included in the pooled estimate.

In addition, we explored predictors of CZ-level association sizes using univariate meta-regression with zone-level exposure and demographic predictors in an attempt to explain heterogeneity in those association sizes using the aforementioned “metareg” Stata package (Harbord and Higgins 2008). We reported variables associated with effect size (*p* < 0.10) after testing mean ozone, mean apparent temperature, mean distance to the monitor, percent race/ethnic group (non-Hispanic White, non-Hispanic Black, Hispanic, non-Hispanic Asian), percent age group (0–18, 65 and older), percent visits within 10 km of an ozone monitor, and binary coastal/noncoastal and Northern/Southern variables.

## Results

Ozone levels varied by climate zone (Table 1). Weighted mean daily 1-hr maximums for visit days were lowest in the coastal, northern climate zone 1 [33 ppb, interquartile range (IQR) = 12] and highest in the arid, inland climate zone 10 (55 ppb, IQR = 29). These gradations were more evident in the warm season, when mean levels for CZs ranged from 31 to 75 ppb (see Table S1). Among EDV-specific exposures over the full year, ozone was most strongly correlated with apparent temperature (*r* = 0.63) (Table 2). Correlations with other air pollutants were weaker.

**Table 1:** Means (IQR) for exposures assigned to eligible*^a^* respiratory EDVs in California, 2005–2008.

CZ	Reference city	O_3_ (ppb)^*b*^		Case-referent difference in O_3_ (ppb)	Distance to O_3_ monitor (m)	Apparent temperature (°F)^*c*^		CO (ppm)^*b*^		NO_2_ (ppb)^*b*^		SO_2_ (ppb)^*b*^	
		All year	Warm season^*d*^			All year	Warm season	All year	Warm season	All year	Warm season	All year	Warm season
1	Arcata	33 (12)	31 (10)	7	6,146 (11,239)	47.2 (11.3)	54.1 (6.4)	0.6 (0.5)	0.4 (0.2)	10 (8)	7 (5)	1 (1)	0 (0)
2	Santa Rosa	36 (11)	40 (14)	9	6,374 (9,217)	49.5 (13.7)	58.2 (8.5)	0.8 (0.7)	0.5 (0.3)	22 (13)	17 (10)	4 (4)	5 (4)
3	Oakland	35 (12)	36 (12)	9	7,425 (8,929)	52.6 (10.3)	58.8 (6.0)	0.9 (0.6)	0.6 (0.4)	26 (18)	21 (15)	3 (4)	3 (2)
4	Sunnyvale	39 (15)	46 (17)	10	6,549 (5,665)	54.1 (13.2)	62.4 (8.1)	1.2 (1.2)	0.7 (0.5)	30 (15)	25 (15)	-	-
5	Santa Maria	40 (10)	40 (11)	7	4,529 (5,200)	52.8 (9.3)	57.7 (5.6)	0.5 (0.4)	0.4 (0.2)	15 (13)	11 (9)	5 (3)	4 (3)
6	Los Angeles	46 (14)	52 (13)	9	7,605 (6,573)	58.7 (11.3)	65.7 (8.2)	0.9 (1.0)	0.5 (0.5)	28 (21)	22 (16)	4 (5)	4 (4)
7	San Diego	49 (13)	53 (13)	10	7,056 (5,337)	59.0 (12.2)	67.0 (8.5)	1.4 (1.2)	1.0 (0.7)	33 (21)	25 (17)	5 (3)	5 (2)
8	El Toro	42 (20)	51 (19)	10	8,257 (6,903)	60.8 (12.7)	68.9 (8.7)	1.5 (1.4)	0.9 (0.7)	40 (19)	34 (20)	4 (4)	4 (4)
9	Pasadena	48 (25)	64 (26)	13	6,905 (4,203)	60.1 (14.8)	69.5 (11.8)	1.2 (1.1)	0.9 (0.6)	40 (19)	38 (19)	2 (2)	2 (2)
10	Riverside	55 (29)	75 (34)	14	7,678 (6,118)	60.0 (17.0)	71.1 (13.5)	1.2 (0.9)	0.9 (0.6)	38 (20)	36 (23)	3 (2)	3 (2)
11	Red Bluff	44 (20)	57 (23)	11	5,742 (5,556)	55.4 (20.0)	69.3 (14.7)	0.9 (0.7)	0.6 (0.4)	24 (19)	23 (20)	1 (1)	2 (1)
12	Sacramento	44 (20)	57 (23)	12	7,091 (6,386)	55.0 (17.3)	66.7 (12.1)	0.9 (0.7)	0.6 (0.4)	25 (16)	22 (16)	3 (2)	3 (2)
13	Fresno	50 (31)	70 (26)	14	6,554 (6,720)	57.4 (20.5)	72.0 (15.7)	1.0 (0.8)	0.7 (0.5)	30 (18)	29 (21)	2 (1)	2 (2)
14	China Lake	54 (25)	70 (27)	10	6,693 (7,953)	54.6 (21.0)	68.8 (16.4)	0.7 (0.7)	0.5 (0.4)	36 (18)	38 (23)	2 (2)	2 (2)
15	El Centro	54 (23)	68 (26)	11	6,379 (8,881)	65.9 (22.9)	82.8 (18.7)	0.8 (0.6)	0.5 (0.3)	29 (20)	24 (20)	2 (3)	2 (3)
16	Mt. Shasta	53 (20)	65 (28)	11	4,625 (6,626)	46.7 (19.8)	57.5 (15.7)	1.1 (0.9)	0.8 (0.5)	27 (35)	27 (36)	-	-
Abbreviations: —, no eligible EDVs with SO_2_ measures present. ^***a***^Having a population-weighted ZIP code centroid located within 20 km of an ozone monitor and 10 km of a temperature monitor. ^***b***^Measures provided by CARB (2011). ^***c***^Measures provided by U.S. EPA (2009), National Climatic Data Center (2011), and California Department of Water Resources (2010). ^***d***^May–October.													

**Table 2:** Correlations for exposures assigned to eligible respiratory EDVs*^a^* in California, 2005–2008.

	Maximum daily 1-hr O_3_ (ppb)^*b*^	Mean daily apparent temperature (°F)^*c*^	Maximum daily 1-hr CO (ppm)^*b*^	Maximum daily 1-hr NO_2_ (ppb)^*b*^	Maximum daily 1-hr SO_2_ (ppb)^*b*^
Full year data
Maximum daily 1-hr O_3_ (ppb)	1	0.63	–0.28	–0.01	–0.06
Mean apparent temperature (°F)		1	–0.17	0.06	0.07
Maximum daily 1-hr CO (ppm)			1	0.62	0.27
Maximum daily 1-hr NO_2_ (ppb)				1	0.24
Maximum daily 1-hr SO_2_ (ppb)					1
Warm season (May–October) data
Maximum daily 1-hr O_3_ (ppb)	1	0.57	0.02	0.26	0.02
Mean apparent temperature (°F)		1	0.04	0.25	0.11
Maximum daily 1-hr CO (ppm)			1	0.66	0.22
Maximum daily 1-hr NO_2_ (ppb)				1	0.21
Maximum daily 1-hr SO_2_ (ppb)					1
Abbreviations: 1-hr, 1 hour; ppb, parts per billion; ppm, parts per million. ^***a***^Having a population-weighted ZIP code centroid located within 20 km of a monitor for the gas pollutants, 10 km for temperature. ^***b***^Measures provided by CARB (2011). ^***c***^Measures provided by U.S. EPA (2009), National Climatic Data Center (2011), or California Department of Water Resources (2010).					

Our analysis included data for 3,654,042 eligible respiratory ED visits (or hospitalizations following an ED visit), with the number of visits by CZ ranging from 4,984 (CZ 1, the northern coastal region) to 590,930 (CZ 9, inland Los Angeles area). Table 3 presents EDV statistics by selected outcomes and demographics. Eligible EDVs occurred less often in the warm season. Females composed the majority of visits, as did private insurance holders. Most ED visitors (75%) lived within 10 km of an ozone monitor. Visitors were ethnically diverse, but proportions varied widely by CZ (see Table S2). Ninety-one percent of visits with eligible ozone information also had NO_2_ exposure available, 83% had CO exposures available, and 54% had SO_2_ available (see Table S3). Populations excluded from co-pollutant analyses were very similar on a number of dimensions, with the only clear difference being a greater proportion of White non-Hispanics and fewer Hispanics and Black non-Hispanics in excluded populations as compared to the full dataset.

**Table 3:** Demographics for eligible respiratory EDV visits within 20 km of an ozone monitor and 10 km of a temperature monitor in California, 2005–2008 (%).

Outcome	Respiratory	ARI	Asthma	Pneumonia	COPD	URTI
ICD-9	(460–519)	(460–466)	(493)	(480–486)	(490–492, 494–496)	(472–473, 476–477)
Total (*N*)	3,654,042	1,696,761	556,168	529,381	196,922	86,678
Season
Warm (May–October)	39	37	44	37	38	44
Age
0–4	29	43	17	23	10	10
5–18	16	20	25	7	7	17
19–64	38	33	49	30	47	67
≥ 65	17	4	9	39	35	6
Race/ethnicity						
White Non-Hispanic	37	30	32	47	52	37
Black Non-Hispanic	12	11	20	9	12	14
Asian Non-Hispanic	4	3	4	7	4	3
Hispanic	38	47	34	31	25	35
Sex
Male	48	48	46	51	45	42

**Figure 2:**
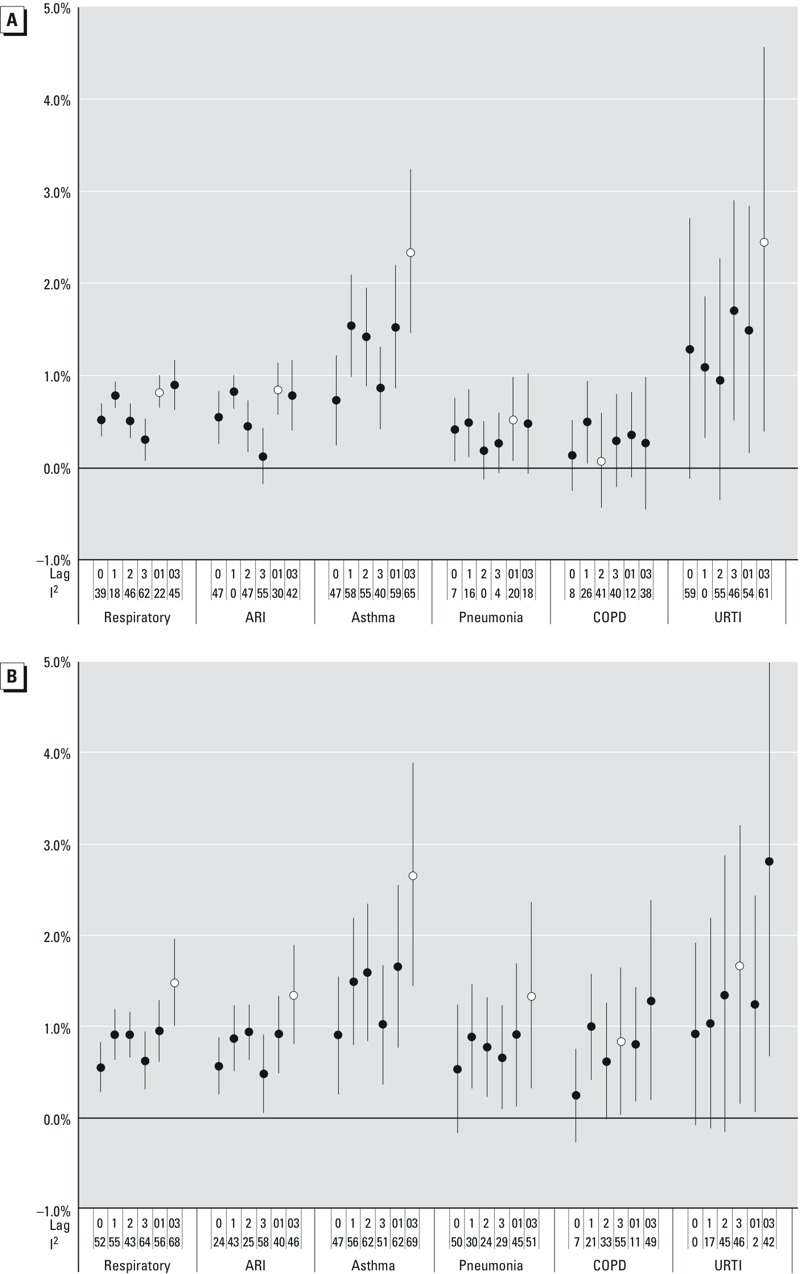
Excess risks (95% CI) per 10-ppb ozone for respiratory outcomes by lag, with *I^2^*statistic, for (*A*) full year, and (*B*) warm season. l = lag of best fit, as identified as the lowest sum of AICs over all CZ analyses. Lag_0_ = same-day exposure, lag_1_ = exposure 1 day prior, etc. Lag_01_ = mean of lag_0_ and lag_1_, lag_03_ = mean of lags 0 through 3, etc. Models adjusted for apparent temperature (lag_0_ and lag_13_) and influenza visits. *I^2^* = [(Q – df)/Q] × 100. Full year = all months available; warm = limited to May through October. Reported risks [(OR – 1) × 100] are pooled estimates using random effects meta-analysis from CZ-specific estimates obtained using conditional logistic regression comparing exposures on visit days with others of the same day of the week within the same month, adjusting for apparent temperature (lag_0_ and lag_13_) and county influenza visits.

In full year analyses, significant associations (*p* < 0.05) were observed between combined respiratory EDVs and ozone levels at multiple lags (Figure 2A), with the model fitting best using lag_01_ ozone (ER_lag01_ = 0.8%, 95% CI: 0.6, 1.0). Among specific diagnoses of respiratory disease, the relationship was strongest with asthma (ER_lag03_ = 2.3%, 95% CI: 1.5, 3.2) and acute respiratory infections (ER_lag01_ = 0.9%, 95% CI: 0.6, 1.1). Multiple ozone lags showed associations with pneumonia (ER_lag01_ = 0.5%, 95% CI: 0.1, 1.0) and URTI (ER_lag03_ = 2.5%, 95% CI: 0.4, 4.6); only one lag showed a COPD association.

In warm season-specific analyses, significant respiratory, acute respiratory infection (ER_lag03_ = 1.4%, 95% CI: 0.8, 1.9), and asthma associations [ER_lag03_ = 2.7% (95% CI: 1.5, 3.9)] persisted (Figure 2B). Warm season analyses also showed more lags with significant associations for acute respiratory infections, pneumonia [ER_lag03_ = 1.3% (95% CI: 0.3, 2.4)], and COPD [ER_lag3_ = 0.8% (95% CI: 0.0, 1.7)]. For all outcomes, associations at corresponding lags were typically similar or slightly larger in magnitude than full year associations.

**Figure 3:**
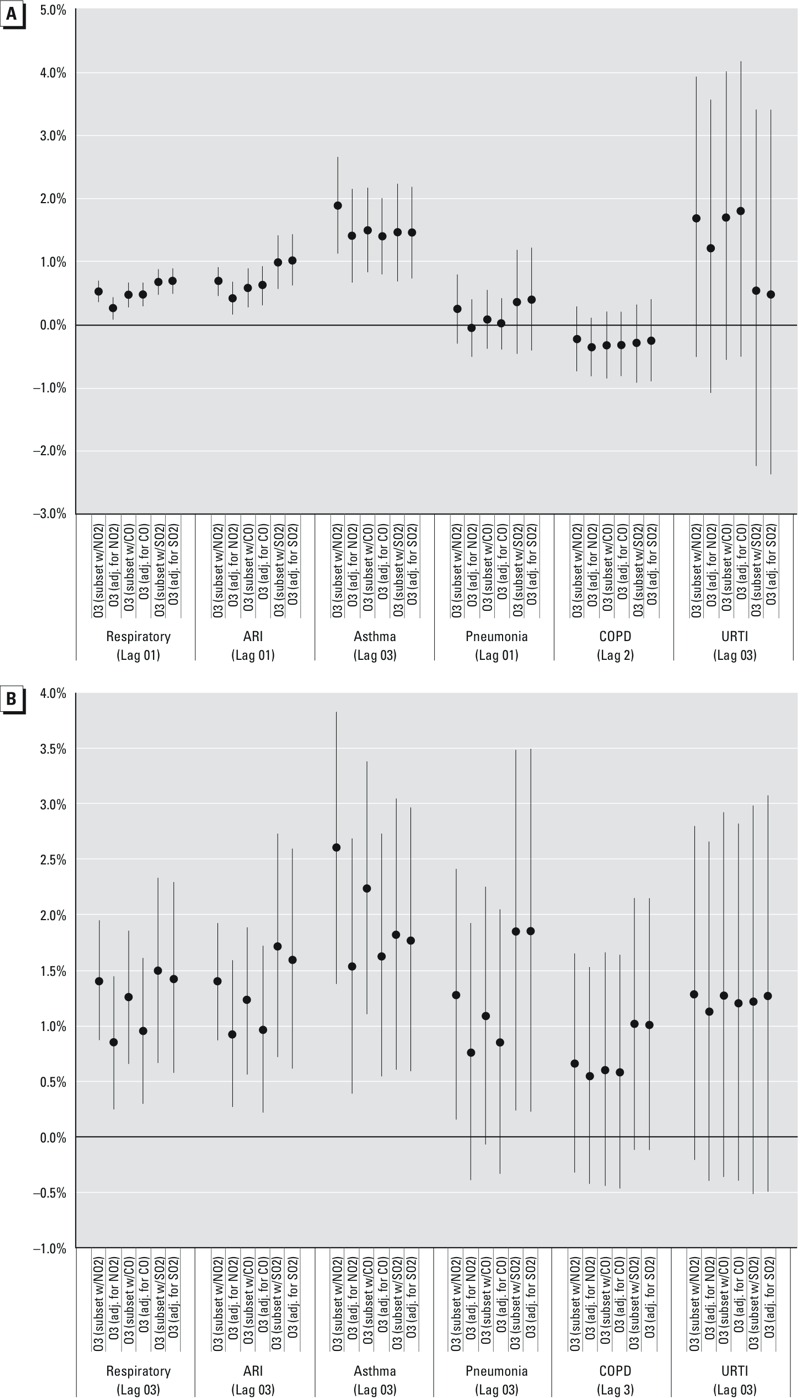
Excess risks (95% CI) per 10-ppb ozone for respiratory outcomes in one- and two-pollutant analyses restricted to the population where another pollutant metric was available for (*A*) full year, (*B*) warm season (May–October). O_3_ (subset w/NO_2_) = models restricted to population with nitrogen dioxide exposures available; O_3_ (adj. for NO_2_) = models with same restricted population but also adjusted for nitrogen dioxide. O_3_ (subset w/CO) = models restricted to population with carbon monoxide exposures available; O_3_ (adj. for CO) = models with same restricted population but also adjusted for nitrogen dioxide. O_3_ (subset w/SO_2_) = models restricted to population with carbon monoxide exposures available; O_3_ (adj. for SO_2_) = models with same restricted population but also adjusted for sulfur dioxide. Reported risks [(OR – 1) × 100] are pooled estimates using random effects meta-analysis from climate zone-specific estimates obtained using conditional logistic regression comparing exposures on visit days with others of the same day of the week within the same month, adjusting for apparent temperature (lag_0_ and lag_13_) and county influenza visits.

In two-pollutant analyses limited to populations where monitoring for a second pollutant was also available within 20 km, for the best fitting lag determined above, we observed the most attenuation of the ozone associations when NO_2_ was added to the model (Figure 3A,B; see also Table S4). Focusing on the warm season, respiratory, ARI, and asthma associations at the best fitting lag remained significantly positive in all two-pollutant models, but the pneumonia effect estimate in the NO_2_-present population was reduced 41% (ER_lag03_ = 0.8%, 95% CI: –0.4, 1.9) and no longer statistically significant. CO-related attenuation was smaller for those outcomes. Warm season COPD and URTI associations shifted little with addition of the other air pollutants, but both one- and two-pollutant results showed a loss of precision and shifts possibly related to population differences in these subsets.

We also explored our COPD case definition. COPD is generally considered a disease of older age, but our ICD-9 range captured cases with only a slight majority (58%) > 50 years of age. Limiting our COPD visits to that age minimum yielded a warm season estimate which was, at its best lag (ER_lag03_ = 1.5%, 95% CI: 0.6, 2.4), similar to, but slightly higher than the original definition at that lag (ER_lag03_ = 1.3%, 95% CI: 0.2, 2.4). The relationships for those visits were somewhat attenuated with NO_2_ adjustment, but loss of precision also influenced the statistical significance of these associations (see Figure S1).

**Figure 4:**
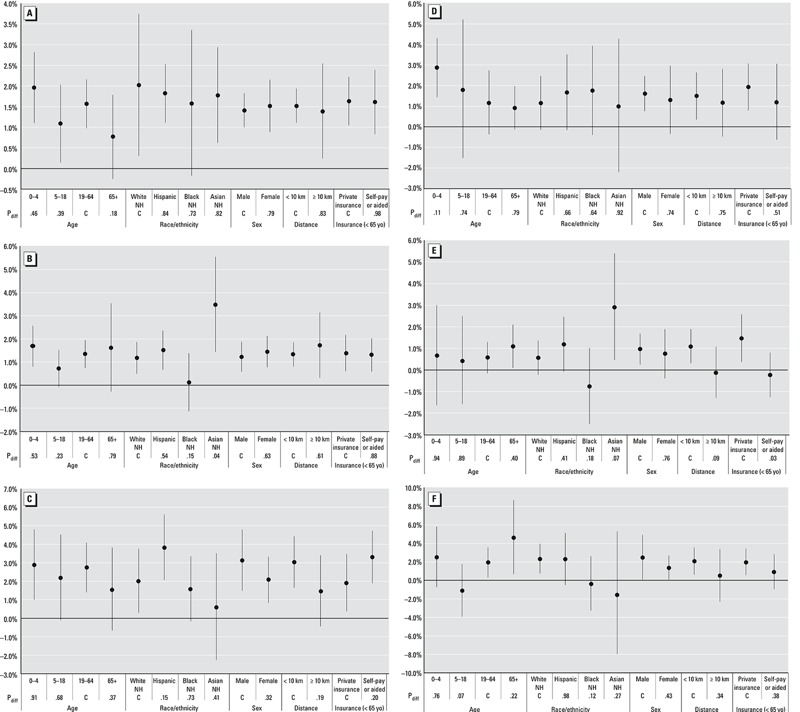
Warm season (May–October) excess risks (95% CI) of EDV per 10-ppb ozone for different demographic/location categories for (*A*) respiratory (lag_03_), (*B*) ARI (lag_03_), (*C*) asthma (lag_03_), (*D*) pneumonia (lag_03_), (*E*) COPD (lag_3_), and (*F*) URTI (lag_3_) types. Models adjusted for apparent temperature (lag_0_ and lag_13_) and influenza outbreaks. Lags based on best fitting lag in non-subset models. Reported risks [(OR – 1) × 100] are pooled estimates using random effects meta-analysis from CZ- and category-specific estimates obtained using conditional logistic regression comparing exposures on visit days with others of the same day of the week within the same month, adjusting for apparent temperature (lag_0_ and lag_13_) and county influenza visits. Note: C, comparison group; km = kilometer; NH = Non-Hispanic; p_diff_ = *p*-value of the difference between the estimates; yo = year olds.

Stratifying by suspected effect modifiers yielded some significant differences between groups, though few were consistent across outcomes. Again, we focused on warm season relationships with an outcome at the best fitting lag identified previously. For these, Asian non-Hispanics showed significantly greater associations with ozone than White non-Hispanics for ARI [ER_lag03_(Asian non-Hispanic) = 3.5%, 95% CI: 1.4, 5.6; ER_lag03_(White non-Hispanic) = 1.2%, 95% CI: 0.5, 1.9; *p*_difference_ = 0.04] and COPD [ER_lag03_(Asian non-Hispanic) = 2.9%, 95% CI: 0.5, 5.4; ER_lag03_(White non-Hispanic) = 0.6%, 95% CI: –0.2, 1.4; *p*_difference_ = 0.07] (Figure 4). Slightly negative estimates for COPD for the self-pay/aid insurance category and those living 10 km or more from a monitor were observed, both being significantly different from those in the self-pay/aid (*p*_difference_ = 0.03) and within 10 km groups (*p*_difference_ = 0.09), respectively. For URTI, children 5–18 years old showed a significantly different association (ER_lag3_ = –1.1%, 95% CI: –3.9, 1.8) compared to adults 19–64 years old (ER_lag3_ = 2.0%, 95% CI: 0.3, 3.6; *p*_difference_ = 0.07). No statistically significant differences between groups were observed for respiratory or pneumonia visits. Taking a broader look over the stratified analysis results, estimates were higher for males in each subcategory other than ARI, with larger differences for asthma [ER_lag03_(males) = 3.1%, 95% CI: 1.5, 4.8; ER_lag03_(females) = 2.1%, 95% CI: 0.8, 3.3; *p*_difference_ = 0.32] and URTI [ER_lag3_(males) = 2.5%, 95% CI: 0.1, 4.9; ER_lag3_(females) = 1.4%, 95% CI: 0.1, 2.7; *p*_difference_ = 0.43]. Hispanics had higher estimates than White non-Hispanics for all subdiagnoses except URTI, whereas Black non-Hispanics had weaker or negative associations for all subdiagnoses except pneumonia. And children 0–4 years of age posted elevated associations compared with adults 19–64 years old for all diagnoses. But none of the individual differences related to these patterns were significantly different.

We observed at least a moderate degree of heterogeneity for some of our outcomes (see Table S5), as seen in the *I^2^* values in Figure 2, implying a multiplicity of true associations influenced by differing population composition and exposure experiences among the zones. When investigating possible explanations for warm season estimates using meta-regression, we found that ozone associations in coastal areas were significantly higher for all respiratory (*p* = 0.01) and COPD (*p* = 0.06) visits, and were generally elevated for pneumonia and asthma as well (see Table S6). Greater mean ozone in the CZ predicted smaller zone-level estimates, significantly so for respiratory, asthma, and COPD. Mirroring individually stratified findings, a higher proportion of Asians in a zone predicted significantly higher zone-level estimates for COPD (*p* = 0.04) and near significantly higher estimates for ARI (*p* = 0.12). Northern location, proportion of 0–18 year olds, of adults ≥ 65 years old, of White non-Hispanics, and of Black non-Hispanics were also significantly associated with association size for one or more of the outcomes.

## Discussion

In this population, we observed associations between ozone exposures and EDVs for respiratory disease, including ARI, asthma, pneumonia, COPD, and URTI. These associations were generally larger in magnitude and present over more lags in the warm season.

A five study meta-analysis of EDV studies published between 1990 and 2008 by Ji et al. (2011) also reported an increase in total respiratory EDVs with increases in ozone after pooling studies and accounting for possible publication bias. Subsequently, a multi-city study of seven Canadian cities found associations with respiratory disease EDVs (3.2%; 95% CI: 0.3, 6.2) (Stieb et al. 2009). Additionally, an Atlanta analysis from 1993 to 2004 also revealed associations between respiratory EDVs, as did a study of St. Louis visits from 2001 to 2007 (Darrow et al. 2011; Winquist et al. 2012).

We observed relationships between ozone exposures and acute respiratory infections and pneumonia visits. Similarly, ozone was associated with increases in EDVs for upper respiratory infection (URI) and pneumonia in Atlanta (Peel et al. 2005). Their associations for URI remained significant when CO and NO_2_ (and, additionally, PM_10_) were included in the models, as ours for ARI did in our subsets with CO and NO_2_ data. Their URI results were stronger in the warm months, but their pneumonia association was higher in the cold months. The seven-city Canadian study investigated, but did not detect, an ozone–respiratory infection association (Stieb et al. 2009).

Our asthma findings comport with the meta-analysis conducted by Ji et al. (2011), which estimated a significant increase between ozone and EDVs for asthma based on a pooled analysis of eight studies, and with studies published subsequently in the United States and Canada (Glad et al. 2012; Gleason et al. 2014; Mar and Koenig 2009; Sacks et al. 2014; Stieb et al. 2009; Strickland et al. 2010; Winquist et al. 2012). Asthma associations observed in our sub-analyses with co-pollutants present remained statistically significant after adjustment by other air pollution measures, similar to studies in Atlanta and New York (Ito et al. 2007; Strickland et al. 2010).

Ozone-COPD EDV studies have been less common, with most studies reporting associations with COPD or COPD/asthma taking place outside the United States (Arbex et al. 2009; Halonen et al. 2008; Kousha and Rowe 2014; Stieb et al. 2009). The pooled effect estimate for emergency COPD hospitalizations in the Ji et al. (2011) meta-analysis was also significantly positive. Future studies should evaluate more specific diagnoses and other risk factors, such as age, that might best identify this group.

Existing epidemiological studies of ozone and upper respiratory tract inflammation have focused on allergic rhinitis in children, which have reported weak or strong positive associations (Hajat et al. 2001; Hwang et al. 2006; Kim BJ et al. 2011). Additionally, occupational ozone exposures have been linked to rhinitis (Hoffman et al. 2004; Karakatsani et al. 2010). Our study, along with these studies, suggests that upper respiratory effects of ozone merit further investigation.

Both our study and previous studies explored demographic-specific associations between ozone and respiratory EDVs. A study of chronic ozone exposures and asthma EDVs in New York City observed elevated associations among Hispanics (Lin et al. 2008). Our study also observed higher magnitudes of associations in this group, though those differences were not statistically significant, and found other race/ethnicity association differences that were statistically significant. CZ estimates also varied significantly depending on proportion of race/ethnicity for some outcomes. This may be related to differences in socioeconomic status, which relate to both differences in exposures related to places of residence (Hackbarth et al. 2011), types of employment, and social behaviors, or differing vulnerability from pre-existing medical conditions, health-related behaviors, higher stress, or other factors (O’Neill et al. 2003; Yip et al. 2011). Access to care may affect susceptibility; insurance status was a statistically significant modifier for COPD in our study, but unexpectedly showing higher associations in those with health insurance. Given that we observed some heterogeneity in associations by race/ethnicity, and that existing evidence provides only weak support of its modification of an ozone effect (Bell et al. 2014), future studies would help confirm whether these ozone–ethnicity differences are true, and what policies might effectively address such disparities. The largest difference in association by sex that we observed was a larger, but not significantly greater, association for asthma in males. However, some previous studies have noted women to suffer greater ozone risks, perhaps due to differences in the structure and morphology of the respiratory system (Bell et al. 2014).

Effect estimates for COPD were significantly higher for those living closer to the monitor, and generally higher for other outcomes as well. An explanation could be that increasing exposure misclassification with increasing distance from the monitor to the residential location is affecting the magnitude of associations observed. Previous studies found ozone values to correlate well over large distances, but small-area differences may be present enough to affect health studies (Bell 2006). Modeling may improve estimates of individual exposures, and may be worth the effort to enhance health effect estimates (Bell 2006). The lack of difference for ARI may relate to the infectious component, which more involves spatial and temporal elements for transmission.

Biological mechanisms by which ozone can cause respiratory distress have been studied in some detail (Mudway and Kelly 2000). Ozone’s strong oxidative potential reacts with extracellular lining fluid to produce secondary oxidation products (Pryor et al. 1995), which initiates a cascade of events involving allergic and inflammatory responses (U.S. EPA 2013). Ultimately, consequences of these events may relate to the induction of airway hyperresponsiveness and predisposition to allergic responses involved in ozone-related asthma visits (Stanek et al. 2011). It may relate to impaired lung host defenses, leaving those exposed more prone to infections (Ciencewicki and Jaspers 2007) involved in our ERVs. Additionally, ozone can inhibit inspiratory depth and promote airway constriction, impacting overall lung function (Kim CS et al. 2011; Stanek et al. 2011). These impacts can play a role in any of the respiratory tract outcomes we found to be associated with ozone exposure.

There are some limitations in our analyses. Our exposure assessment focuses on residence-proximate ozone measurements, and, to reduce misclassification we only analyzed people living near ozone monitors. However, people do not spend all of their time at home. Other factors, such as time spent outdoors and degree of ozone penetration indoors, also influence a person’s actual exposure. We observed larger associations in coastal climate zones, perhaps as a result of less home air conditioning and thus greater warm season ventilation from outdoors in these areas (Ostro et al. 2010). Our analysis also does not account for behavioral changes related to outdoor ozone levels. For example, schools in high pollution areas may limit outdoor physical activities on days of high pollution. Consequently, we might observe weaker relationships due to reduced personal exposures on days where ambient monitors would predict greater exposures. Our meta-regression findings of lesser associations in higher ozone communities may reflect this to some extent. Exposures for patients ascertained via hospital discharge records were classified based on date of hospital admission, which may have actually occurred the day after the ED visit that led to the admission. We were also not able to identify multiple visits by the same individuals, which would violate the assumption of independent observations. Our referent periods best account for day of week differences. However, we were unable to evaluate PM_2.5_ as a confounder due to its incompatible monitoring schedule. PM_2.5_ was not strongly correlated with ozone in California over the entire year during this time period (median monitor correlation = 0.01), reducing its likelihood as a confounder in those analyses, but may influence summer only analyses (median correlation = 0.36) (CARB 2011). Further study is needed to explore its role in these relationships.

In summary, ozone showed associations with asthma, ARI, COPD, pneumonia, and URTI visits in the warm season. Studies examining the health benefits of ozone reductions should try to account for ozone-EDV relationships to get a fuller picture of those benefits.

## Supplemental Material

(504 KB) PDF
