# Supplementary material for: A Time-Stratified Case-Crossover Study of Ambient Ozone Exposure and Emergency Department Visits for Specific Respiratory Diagnoses in California (2005–2008)
Source: Environ Health Perspect. 2015 Dec 8;124(6):745–53. doi: 10.1289/ehp.1409495 (PMC4892911; doi:10.1289/ehp.1409495)

Environ Health Perspect
DOI: 10.1289/ehp.1409495

Note to readers with disabilities: *EHP* strives to ensure that all journal content is accessible to all readers. However, some figures and Supplemental Material published in *EHP* articles may not conform to 508 standards due to the complexity of the information being presented. If you need assistance accessing journal content, please contact ehp508@niehs.nih.gov. Our staff will work with you to assess and meet your accessibility needs within 3 working days.

Supplemental Material

A Time-Stratified Case-Crossover Study of Ambient Ozone Exposure and Emergency Department Visits for Specific Respiratory Diagnoses in California (2005-2008)

Brian J. Malig, Dharshani L. Pearson, Yun Brenda Chang, Rachel Broadwin, Rupa Basu, Rochelle S. Green, and Bart Ostro

Table of Contents

Table S1. Geographic indicators and exposure variable means by climate zone for eligible[a] respiratory EDVs in California, 2005-2008.

Table S2. Demographics by climate zone for eligible respiratory EDVs in California, 2005-2008.

Table S3. Demographic comparisons of eligible respiratory EDVs for those with ozone exposures assigned compared to those missing available co-pollutant exposure data with 20km in California, 2005-2008. NH = non-Hispanic; k = km;

Table S4. Excess risks (95% CI) per 10ppb ozone for respiratory outcomes in one- and two-pollutant analyses restricted to the population where another pollutant metric was available for full year and warm season (May-October). Reported risks [(OR-1)*100] are pooled estimates using random effects meta-analysis from climate zone-specific estimates obtained using conditional logistic regression comparing exposures on visit days with others of the same day of

the week within the same month, adjusting for apparent temperature (lag_0 and lag_{1-3}) and county influenza visits.

Table S5. Climate zone-level effect estimates and standard errors for warm season (May-October) respiratory EDV types in California, 2005-2008, using the best fitting lag.

Table S6. Meta-regression coefficients for warm season (May- October) respiratory EDV types and climate zone-level variables in California, 2005-2008.

Figure S1. Excess risks (95% CI) per 10ppb ozone (lag 03) for COPD limited to persons 50 years of age and above, in one- and two-pollutant analyses restricted to the population where another pollutant metric was available for (A) full year, (B) warm season (May-October). O_3=no subset; O_3 (subset w/NO_2) = models restricted to population with nitrogen dioxide exposures available; O_3 (adj. for NO_2) = models with same restricted population but also adjusted for nitrogen dioxide. O_3 (subset w/CO) = models restricted to population with carbon monoxide exposures available; O_3 (adj. for CO) = models with same restricted population but also adjusted for nitrogen dioxide. O_3 (subset w/SO_2) = models restricted to population with carbon monoxide exposures available; O_3 (adj. for SO_2) = models with same restricted population but also adjusted for sulfur dioxide. Reported risks [(OR-1)*100] are pooled estimates using random effects meta-analysis from climate zone-specific estimates obtained using conditional logistic regression comparing exposures on visit days with others of the same day of the week within the same month, adjusting for apparent temperature (lag_0 and lag_{13}) and county influenza visits.

Table S1. Geographic indicators and exposure variable means by climate zone for eligible[a] respiratory EDVs in California, 2005-2008.

	Location variables		Exposure-related variables		
CZ	Coastal	Northern	Mean warm season ozone (ppm)[b]	Mean warm season apparent temperature (°F)[c]	Mean distance to ozone monitor (m)
1	1	1	0.031222	54.07840282	6146.231333
2	0	1	0.039758	58.22339114	6373.887884
3	1	1	0.036429	58.80977328	7425.272865
4	0	1	0.046496	62.39072904	6548.714634
5	1	0	0.039672	57.68523216	4528.765443
6	1	0	0.051985	65.7171559	7604.505967
7	1	0	0.052503	67.02876139	7055.639389
8	0	0	0.051223	68.88763884	8257.005309
9	0	0	0.064468	69.48360102	6904.955864
10	0	0	0.07475	71.07552369	7677.779765
11	0	1	0.057432	69.2735873	5741.746846
12	0	1	0.056818	66.66581458	7091.026887
13	0	1	0.069696	71.98453568	6553.566692
14	0	0	0.0701	68.7765554	6692.637303
15	0	0	0.067806	82.83219023	6378.538403
16	0	-	0.064918	57.54531365	4625.26612

[a]having a population weighted zip code centroid located within 20km of an ozone monitor and 10km of a temperature monitor

[b]measures provided by California Air Resources Board

[c]measures provided by US EPA, National Climatic Data Center, and California Irrigation Management Information System

Table S2. Demographics by climate zone for eligible[a] respiratory EDVs in California, 2005-2008.

	Age (proportion)		Race/ethnicity (proportion)				Distance (proportion)	
CZ	0-18	65+	White	Black	Hispanic	Asian	< 10k	>= 10k
1	0.269060995	0.203250401	0.846508828	0.025280899	0.045545746	0.016051364	0.721107544	0.278892456
2	0.341121055	0.215367405	0.647197362	0.027614225	0.233643429	0.015014131	0.690390956	0.309609044
3	0.376832163	0.180183343	0.303336027	0.189784838	0.272566796	0.093502197	0.708250417	0.291749583
4	0.417991779	0.191235329	0.384823409	0.039165179	0.377839526	0.109849099	0.850039213	0.149960787
5	0.444335459	0.193165099	0.519505997	0.029983503	0.385371617	0.016251282	0.827566989	0.172433011
6	0.367857067	0.243299144	0.532023634	0.046526567	0.284898455	0.057835255	0.722591257	0.277408743
7	0.395778881	0.199495676	0.4048118	0.118896015	0.342053449	0.055659823	0.813446326	0.186553674
8	0.527799873	0.138262348	0.218772307	0.202064757	0.463485724	0.03439408	0.67003191	0.32996809
9	0.463146395	0.185640001	0.302129101	0.064149122	0.503119972	0.05333593	0.824946222	0.175053778
10	0.48508081	0.134145428	0.342235433	0.133359288	0.384080541	0.018273387	0.745752348	0.254247652
11	0.342703855	0.225811193	0.809971337	0.021671628	0.107549701	0.016600522	0.813140778	0.186859222
12	0.386761028	0.17806663	0.498581426	0.129108037	0.251836441	0.041257197	0.742177097	0.257822903
13	0.522410947	0.124061778	0.325606222	0.08815804	0.478829011	0.019329396	0.746747863	0.253252137
14	0.510911465	0.112198499	0.417672326	0.193055053	0.30784178	0.008386173	0.707309974	0.292690026
15	0.53690404	0.157138255	0.300860305	0.045004927	0.617429698	0.004292049	0.693094823	0.306905177
16	0.371370623	0.199829206	0.752775406	0.009500427	0.099914603	0.003842869	0.82087959	0.17912041

[a]having a population weighted zip code centroid located within 20km of an ozone monitor and 10km of a temperature monitor

Table S3. Demographic comparisons of eligible[a] respiratory EDVs for those with ozone exposures assigned compared to those missing available co-pollutant exposure data with 20km in California, 2005-2008. NH = non-Hispanic; k = km;

	**All**	**Missing NO_2**	**Missing CO**	**Missing SO_2**
	N = 3,654,042	N = 333,935	N = 613,265	N = 1,680,896
**Continuous variables (mean)**				
Ozone (ppm)[b]	0.045	0.046	0.047	0.046
Distance to Ozone monitor (m)	7159	6935	7188	7113
**Categorical variables (%)**				
Age				
0-4	29	28	28	28
5-18	16	16	16	16
19-64	38	39	38	38
65+	17	17	17	17
Race/ethnicity				
White NH	37	53	51	44
Black NH	12	7	6	8
Hispanic	38	30	33	35
Asian NH	4	1	2	4
Distance to ozone monitor				
< 10k	75	72	72	74
10-20k	25	28	28	26
Season				
Cool	61	60	60	62
Warm	39	40	40	38
Sex				
Male	48	47	47	48
Female	52	53	53	52
Expected Payment Method				
Private Insurance	61	58	58	60
Self-pay/Aided	39	42	42	40

[a]having a population weighted zip code centroid located within 20km of an ozone monitor and 10km of a temperature monitor

[b]measures provided by California Air Resources Board

Table S4. Excess risks (95% CI) per 10ppb ozone for respiratory outcomes in one- and two-pollutant analyses restricted to the population where another pollutant metric was available for full year and warm season (May-October). Reported risks [(OR-1)*100] are pooled estimates using random effects meta-analysis from climate zone-specific estimates obtained using conditional logistic regression comparing exposures on visit days with others of the same day of the week within the same month, adjusting for apparent temperature (lag_0 and lag_{1-3}) and county influenza visits.

		ALL YEAR				WARM SEASON			
Outcome/Lag	Model	Number of Climate Zones	ERper10ppb	LCLfor10ppb	UCLfor10ppb	Number of Climate Zones	ERper10ppb	LCLfor10ppb	UCLfor10ppb
Respiratory (Lag 01)	O_3 (subset w/NO_2)	16	0.54%	0.37%	0.70%	16	1.40%	0.87%	1.94%
	O_3 (adj. for NO_2)	16	0.27%	0.10%	0.44%	16	0.85%	0.25%	1.45%
	O_3 (subset w/CO)	16	0.48%	0.30%	0.66%	16	1.26%	0.66%	1.86%
	O_3 (adj. for CO)	16	0.48%	0.30%	0.67%	16	0.95%	0.30%	1.60%
	O_3 (subset w/SO_2)	14	0.68%	0.48%	0.87%	14	1.50%	0.67%	2.33%
	O_3 (adj. for SO_2)	14	0.70%	0.50%	0.89%	14	1.43%	0.57%	2.29%
ARI (Lag 01)	O_3 (subset w/NO_2)	16	0.69%	0.47%	0.91%	16	1.40%	0.88%	1.92%
	O_3 (adj. for NO_2)	16	0.42%	0.17%	0.68%	16	0.93%	0.27%	1.59%
	O_3 (subset w/CO)	16	0.59%	0.29%	0.88%	16	1.23%	0.57%	1.88%
	O_3 (adj. for CO)	16	0.62%	0.32%	0.93%	16	0.97%	0.22%	1.72%
	O_3 (subset w/SO_2)	14	1.00%	0.58%	1.41%	14	1.72%	0.72%	2.73%
	O_3 (adj. for SO_2)	14	1.02%	0.62%	1.43%	14	1.60%	0.61%	2.60%
Asthma (Lag 03)	O_3 (subset w/NO_2)	16	1.89%	1.14%	2.66%	16	2.60%	1.39%	3.83%
	O_3 (adj. for NO_2)	16	1.41%	0.68%	2.15%	16	1.54%	0.39%	2.69%
	O_3 (subset w/CO)	16	1.50%	0.85%	2.16%	16	2.24%	1.11%	3.38%
	O_3 (adj. for CO)	16	1.41%	0.81%	2.00%	16	1.63%	0.54%	2.73%
	O_3 (subset w/SO_2)	14	1.46%	0.69%	2.23%	14	1.82%	0.61%	3.04%
	O_3 (adj. for SO_2)	14	1.46%	0.75%	2.17%	14	1.77%	0.59%	2.96%

Pneumonia									
(Lag 01)	O_3 (subset w/NO_2)	16	0.25%	-0.28%	0.79%	16	1.28%	0.16%	2.41%
	O_3 (adj. for NO_2)	16	-0.05%	-0.50%	0.40%	16	0.76%	-0.39%	1.92%
	O_3 (subset w/CO)	16	0.09%	-0.36%	0.55%	16	1.09%	-0.06%	2.25%
	O_3 (adj. for CO)	16	0.02%	-0.38%	0.42%	16	0.85%	-0.33%	2.05%
	O_3 (subset w/SO_2)	14	0.36%	-0.45%	1.18%	14	1.85%	0.24%	3.49%
	O_3 (adj. for SO_2)	14	0.40%	-0.40%	1.21%	14	1.85%	0.23%	3.49%
COPD									
(Lag 2)	O_3 (subset w/NO_2)	16	-0.23%	-0.73%	0.28%	16	0.66%	-0.31%	1.65%
	O_3 (adj. for NO_2)	16	-0.35%	-0.80%	0.11%	16	0.55%	-0.42%	1.52%
	O_3 (subset w/CO)	16	-0.32%	-0.84%	0.21%	16	0.60%	-0.44%	1.66%
	O_3 (adj. for CO)	16	-0.31%	-0.81%	0.20%	16	0.58%	-0.46%	1.63%
	O_3 (subset w/SO_2)	14	-0.29%	-0.90%	0.32%	14	1.01%	-0.11%	2.15%
	O_3 (adj. for SO_2)	14	-0.25%	-0.89%	0.40%	14	1.01%	-0.11%	2.15%
URTI									
(Lag 03)	O_3 (subset w/NO_2)	16	1.69%	-0.49%	3.92%	16	1.28%	-0.21%	2.80%
	O_3 (adj. for NO_2)	16	1.22%	-1.07%	3.56%	16	1.12%	-0.39%	2.66%
	O_3 (subset w/CO)	16	1.71%	-0.54%	4.02%	16	1.27%	-0.36%	2.92%
	O_3 (adj. for CO)	16	1.81%	-0.50%	4.17%	16	1.20%	-0.39%	2.82%
	O_3 (subset w/SO_2)	14	0.54%	-2.23%	3.40%	14	1.22%	-0.51%	2.99%
	O_3 (adj. for SO_2)	14	0.49%	-2.36%	3.41%	14	1.27%	-0.50%	3.07%

Table S5. Climate zone-level effect estimates and standard errors for warm season (May-October) respiratory EDV types in California, 2005-2008, using the best fitting lag.

CZ	Respiratory Warm Lag03 Estimate	Respiratory Warm Lag03 StdErr	ARI Warm Lag03 Estimate	ARI Warm 03 StdErr	Asthma Warm Lag03 Estimate	Asthma Warm Lag03 StdErr	Pneumonia Warm Lag03 Estimate	Pneumonia Warm Lag03 StdErr	COPD Warm Lag3 Estimate	COPD Warm Lag3 StdErr	URTI Warm Lag3 Estimate	URTI Warm Lag3 StdErr
1	0.1204142	0.078208	-0.09122	0.139253	0.141563	0.182059	0.437618	0.217531	0.34174	0.137639	-0.17549	0.301123
2	0.0326262	0.009532	0.024566	0.015175	0.052533	0.024196	0.037153	0.024135	0.00191	0.019609	0.018623	0.037177
3	0.0112102	0.004875	0.004921	0.007583	0.022973	0.011037	0.009001	0.013074	0.013307	0.010428	0.026687	0.01926
4	0.0197667	0.006324	0.022438	0.009434	0.037454	0.015897	0.001704	0.016005	0.029024	0.014199	0.04847	0.027194
5	0.0368995	0.017123	-0.00211	0.024898	0.059733	0.048209	0.11301	0.047705	0.075047	0.033704	-0.07351	0.078111
6	0.0292997	0.007133	0.045921	0.011443	0.030213	0.018179	0.005433	0.017737	0.003254	0.014311	-0.00264	0.033513
7	0.0378146	0.007073	0.036028	0.011303	0.05993	0.016172	0.061857	0.01806	0.038446	0.01322	0.037535	0.032078
8	0.0162113	0.003411	0.016714	0.004935	0.017848	0.008108	0.007343	0.009809	0.011701	0.0073	-0.01474	0.016511
9	0.0140243	0.002382	0.015757	0.00353	0.007863	0.006025	0.022406	0.0064	0.015578	0.005176	0.014507	0.011023
10	0.008453	0.002733	0.008053	0.003934	0.003515	0.006946	0.028021	0.007533	0.001601	0.005669	-0.00017	0.011882
11	0.0061396	0.006673	-0.00476	0.010653	0.067498	0.018568	0.000334	0.016487	-0.01373	0.01209	0.000524	0.028597
12	0.0123685	0.003027	0.010213	0.00478	0.030003	0.007136	0.009696	0.007908	0.003962	0.005783	0.053019	0.011894
13	0.0166414	0.00417	0.019672	0.006196	0.041236	0.010181	-0.00632	0.01125	0.003435	0.008432	0.025384	0.015498
14	-0.0091484	0.006353	0.004952	0.009184	-0.03724	0.016039	-0.014	0.0174	-0.0166	0.012093	-0.03942	0.024092
15	0.0088754	0.006887	-0.00354	0.009372	0.04707	0.019054	-0.00737	0.019804	0.014304	0.013331	0.052034	0.026301
16	-0.0152106	0.020605	-0.01425	0.029972	-0.03028	0.064933	0.000758	0.050108	-0.06879	0.040778	0.082159	0.083881

Table S6. Meta-regression coefficients for warm season (May- October) respiratory EDV types and climate zone-level variables in California, 2005-2008.

Outcome	Predictor	Estimate	Std. Error	p
Respiratory	Coastal (vs. non-coastal)	0.013793	0.005532	0.013
Respiratory	Northern (vs. Southern)	0.000471	0.005089	0.926
Respiratory	Mean warm season ozone (ppm)	-0.48381	0.195262	0.013
Respiratory	Mean distance to ozone monitor (m)	1.27E-06	3.26E-06	0.698
Respiratory	Mean warm season apparent temperature (degF)	-0.00059	0.00042	0.16
Respiratory	less than 10k from monitor (proportion)	0.032654	0.045244	0.47
Respiratory	age 0-18 (proportion)	-0.05649	0.035998	0.117
Respiratory	age 65+ (proportion)	0.152663	0.061449	0.013
Respiratory	White non-Hispanic (proportion)	0.002502	0.016341	0.878
Respiratory	Black non-Hispanic (proportion)	-0.0552	0.03906	0.158
Respiratory	Hispanic (proportion)	0.004944	0.019683	0.802
Respiratory	Asian non-Hispanic(proportion)	0.120181	0.078114	0.124
ARI	Coastal (vs. non-coastal)	0.010723	0.007207	0.137
ARI	Northern (vs. Southern)	-0.00266	0.005906	0.653
ARI	Mean warm season ozone (ppm)	-0.22285	0.246078	0.365
ARI	Mean distance to ozone monitor (m)	5.40E-06	3.89E-06	0.165
ARI	Mean warm season apparent temperature (degF)	-0.00048	0.000514	0.348
ARI	less than 10k from monitor (proportion)	0.024093	0.05223	0.645
ARI	age 0-18 (proportion)	-0.02382	0.04372	0.586
ARI	age 65+ (proportion)	0.097554	0.078801	0.216
ARI	White non-Hispanic (proportion)	-0.01028	0.019923	0.606
ARI	Black non-Hispanic (proportion)	-0.01855	0.047047	0.693
ARI	Hispanic (proportion)	0.008796	0.022925	0.701
ARI	Asian non-Hispanic(proportion)	0.146289	0.09295	0.116
Asthma	Coastal (vs. non-coastal)	0.015637	0.014556	0.283
Asthma	Northern (vs. Southern)	0.022395	0.010716	0.037
Asthma	Mean warm season ozone (ppm)	-0.87741	0.495951	0.077
Asthma	Mean distance to ozone monitor (m)	-1.2E-05	8.21E-06	0.145

Asthma	Mean warm season apparent temperature (degF)	-0.00019	0.001112	0.868
Asthma	less than 10k from monitor (proportion)	0.10873	0.116066	0.349
Asthma	age 0-18 (proportion)	-0.16331	0.086646	0.059
Asthma	age 65+ (proportion)	0.379081	0.158905	0.017
Asthma	White non-Hispanic (proportion)	0.06697	0.039426	0.089
Asthma	Black non-Hispanic (proportion)	-0.21895	0.098475	0.026
Asthma	Hispanic (proportion)	-0.02841	0.049985	0.57
Asthma	Asian non-Hispanic (proportion)	0.094378	0.207141	0.649
Pneumonia	Coastal (vs. non-coastal)	0.020032	0.013033	0.124
Pneumonia	Northern (vs. Southern)	-0.01202	0.010257	0.241
Pneumonia	Mean warm season ozone (ppm)	-0.39345	0.47133	0.404
Pneumonia	Mean distance to ozone monitor (m)	-2.04E-07	7.29E-06	0.978
Pneumonia	Mean warm season apparent temperature (degF)	-0.00118	0.000991	0.232
Pneumonia	less than 10k from monitor (proportion)	0.110993	0.093122	0.233
Pneumonia	age 0-18 (proportion)	-0.08299	0.080223	0.301
Pneumonia	age 65+ (proportion)	0.156746	0.145419	0.281
Pneumonia	White non-Hispanic (proportion)	0.007175	0.035718	0.841
Pneumonia	Black non-Hispanic (proportion)	-0.03357	0.08774	0.702
Pneumonia	Hispanic (proportion)	-0.00865	0.043381	0.842
Pneumonia	Asian non-Hispanic (proportion)	0.040083	0.181665	0.825
COPD	Coastal (vs. non-coastal)	0.018658	0.009989	0.062
COPD	Northern (vs. Southern)	-0.00454	0.008316	0.585
COPD	Mean warm season ozone (ppm)	-0.63213	0.354142	0.074
COPD	Mean distance to ozone monitor (m)	1.82E-06	5.73E-06	0.751
COPD	Mean warm season apparent temperature (degF)	-0.00047	0.000764	0.535
COPD	less than 10k from monitor (proportion)	0.079058	0.075825	0.297
COPD	age 0-18 (proportion)	-0.00428	0.063697	0.946
COPD	age 65+ (proportion)	0.084054	0.113814	0.46
COPD	White (proportion)	-0.03961	0.026403	0.134
COPD	Black non-Hispanic (proportion)	-0.02042	0.069278	0.768
COPD	Hispanic non-Hispanic (proportion)	0.048154	0.031325	0.124
COPD	Asian non-Hispanic (proportion)	0.275258	0.130839	0.035

URTI	Coastal (vs. non-coastal)	-0.00025	0.020765	0.99
URTI	Northern (vs. Southern)	0.031175	0.012099	0.01
URTI	Mean warm season ozone (ppm)	-0.36818	0.673097	0.584
URTI	Mean distance to ozone monitor (m)	-8.69E-06	1.09E-05	0.423
URTI	Mean warm season apparent temperature (degF)	9.73E-05	0.001434	0.946
URTI	less than 10k from monitor (proportion)	0.125047	0.146299	0.393
URTI	age 0-18 (proportion)	-0.14859	0.106961	0.165
URTI	age 65+ (proportion)	0.248985	0.21087	0.238
URTI	White non-Hispanic (proportion)	0.014428	0.054045	0.789
URTI	Black non-Hispanic (proportion)	-0.15983	0.12638	0.206
URTI	Hispanic (proportion)	0.003211	0.063521	0.96
URTI	Asian non-Hispanic (proportion)	0.320169	0.259207	0.217

Figure S1. Excess risks (95% CI) per 10ppb ozone (lag 03) for COPD limited to persons 50 years of age and above, in one- and two-pollutant analyses restricted to the population where another pollutant metric was available for (A) full year, (B) warm season (May-October). O_3=no subset; O_3 (subset w/NO_2) = models restricted to population with nitrogen dioxide exposures available; O_3 (adj. for NO_2) = models with same restricted population but also adjusted for nitrogen dioxide. O_3 (subset w/CO) = models restricted to population with carbon monoxide exposures available; O_3 (adj. for CO) = models with same restricted population but also adjusted for nitrogen dioxide. O_3 (subset w/SO_2) = models restricted to population with carbon monoxide exposures available; O_3 (adj. for SO_2) = models with same restricted population but also adjusted for sulfur dioxide. Reported risks [(OR-1)*100] are pooled estimates using random effects meta-analysis from climate zone-specific estimates obtained using conditional logistic regression comparing exposures on visit days with others of the same day of the week within the same month, adjusting for apparent temperature (lag_0 and lag_{13}) and county influenza visits.

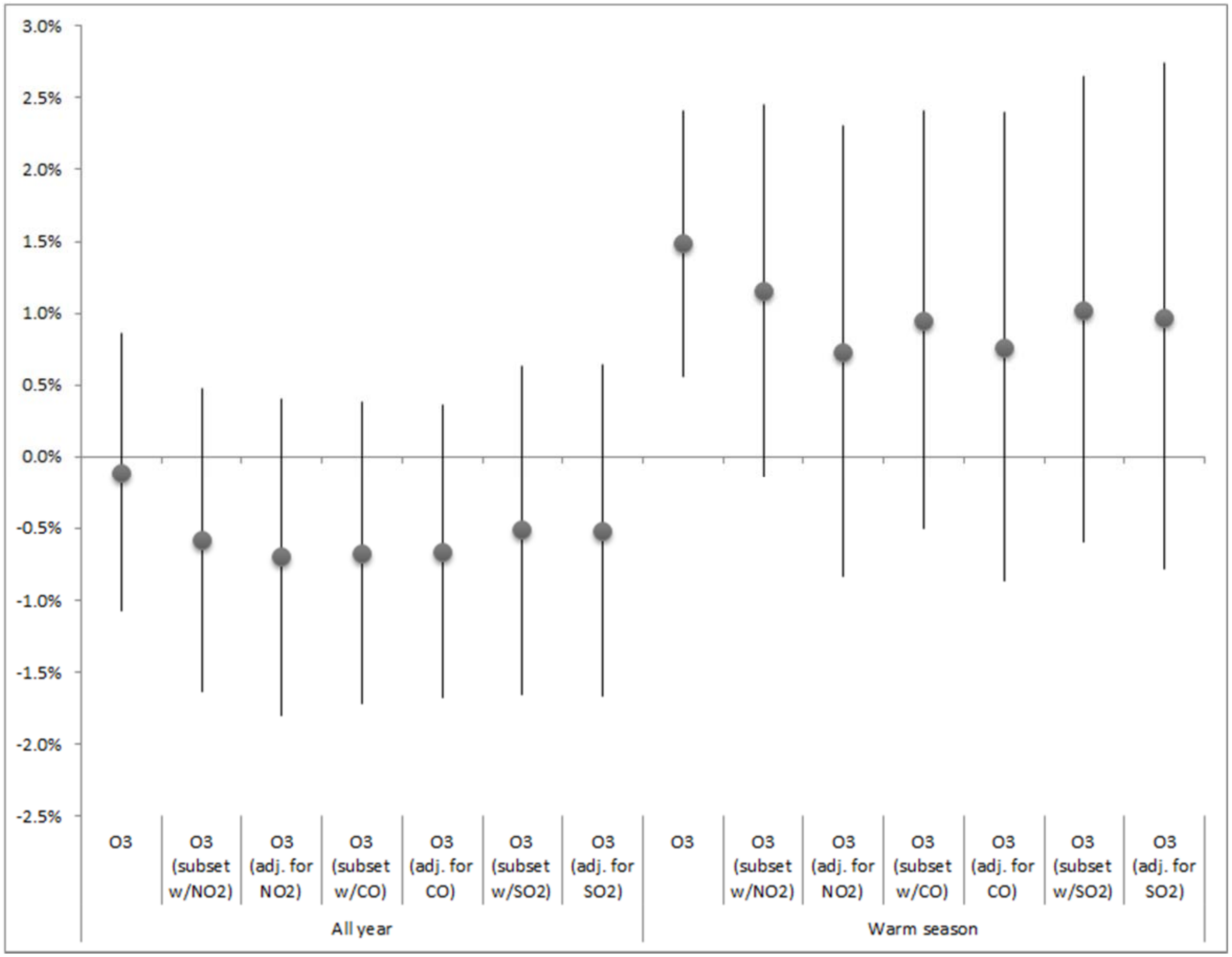

Supplement: (504 KB) PDF [file ehp.1409495.s001.acco.pdf]
